# Gonococcal Septic Arthritis Involving Bilateral Wrists and Hands in an Elderly Patient

**DOI:** 10.7759/cureus.85018

**Published:** 2025-05-29

**Authors:** David K Willett, Evan Siau

**Affiliations:** 1 Medicine, NYU Grossman School of Medicine, New York, USA

**Keywords:** ceftriaxone, dgi, disseminated gonorrhea, dry tap arthrocentesis, gonorrhea, improvement of symptoms with iv ceftriaxone, naat, neisseria gonorrhoeae, polyarthralgias, septic arthritis

## Abstract

*Neisseria gonorrhoeae (N. gonorrhoeae)* is a Gram-negative intracellular diplococcus that is commonly spread via sexual activity. The bacteremic spread of *gonorrhoeae* can lead to an uncommon complication called disseminated gonococcal infection (DGI). DGI has a range of clinical presentations, some of which are vague and nonspecific, including polyarthralgias, isolated septic arthritis, and systemic symptoms. Septic arthritis is a painful joint infection that can lead to inflammation, swelling, and limited mobility. We discuss a case of a male in his mid-70s with sudden bilateral wrist pain and swelling due to a DGI. The diagnosis was complicated by multiple unsuccessful arthrocentesis attempts ("dry taps") but was eventually confirmed by tissue culture, and the patient was treated with ceftriaxone. This report highlights a unique clinical presentation of DGI, the complex nature of its diagnosis, and the importance of thorough history taking.

## Introduction

*Neisseria gonorrhoeae (N. gonorrhoeae)* is a Gram-negative intracellular diplococcus bacterium that causes the sexually transmitted infection (STI) gonorrhea. In 2023, 601,319 gonorrhea cases were reported, making it the second most common STI in the US after chlamydia, per CDC data [1,2]. *N. gonorrhoeae* is transmitted sexually through direct contact with mucous membranes and primarily infects the urinary and genital tracts but can also colonize the anus, nasopharynx, and eyes [1].

Male urethral infections often present with dysuria, testicular pain, and purulent discharge within one week of infection and, if untreated, can lead to acute epididymitis, acute purulent urethritis, and prostatitis [1,3,4]. Between 0.5-3% of patients infected with untreated *N. gonorrhoeae* develop disseminated gonococcal infection (DGI) and manifest a variety of symptoms including migratory polyarthralgias, dermatologic lesions, tenosynovitis, or gonococcal arthritis with or without systemic symptoms [5,6,7]. One of the major clinical syndromes that result from DGI is localized septic arthritis, which occurs in approximately 40% of DGI infections [5,8]. Localized septic arthritis presents with mono-, oligo-, or polyarthritis of multiple joints with associated swelling, pain, and limited range of motion [5,8]. We present a case of a male patient in his mid-70s who was admitted for isolated, bilateral wrists localized gonococcal septic oligoarthritis without systemic symptoms associated with DGI.

## Case presentation

The patient was an elderly African American male with diabetes mellitus type 2, hyperlipidemia, and prostate cancer status post radiation/chemotherapy who presented with bilateral hand and wrist pain, worse on the left side, with associated edema. The wrist pain had started two weeks prior, and there was no history of trauma or recent sexual activity. The patient had bilateral wrist swelling and severe pain that was worse on the left side. He had presented to an urgent care facility and had been referred to a rheumatologist, who suspected severe rheumatoid arthritis (RA) based on an elevated rheumatoid factor level of 41 IU/mL (normal level: under 14 IU/mL) 10 days before the admission. He had been started on daily prednisone (40 mg), weekly methotrexate (20 mg), and an intramuscular (IM) steroid injection of an unknown dose. Despite the treatment, his pain had worsened to an intolerable level, and the patient was admitted nine days after the start of the treatment. 

**Figure 1:**
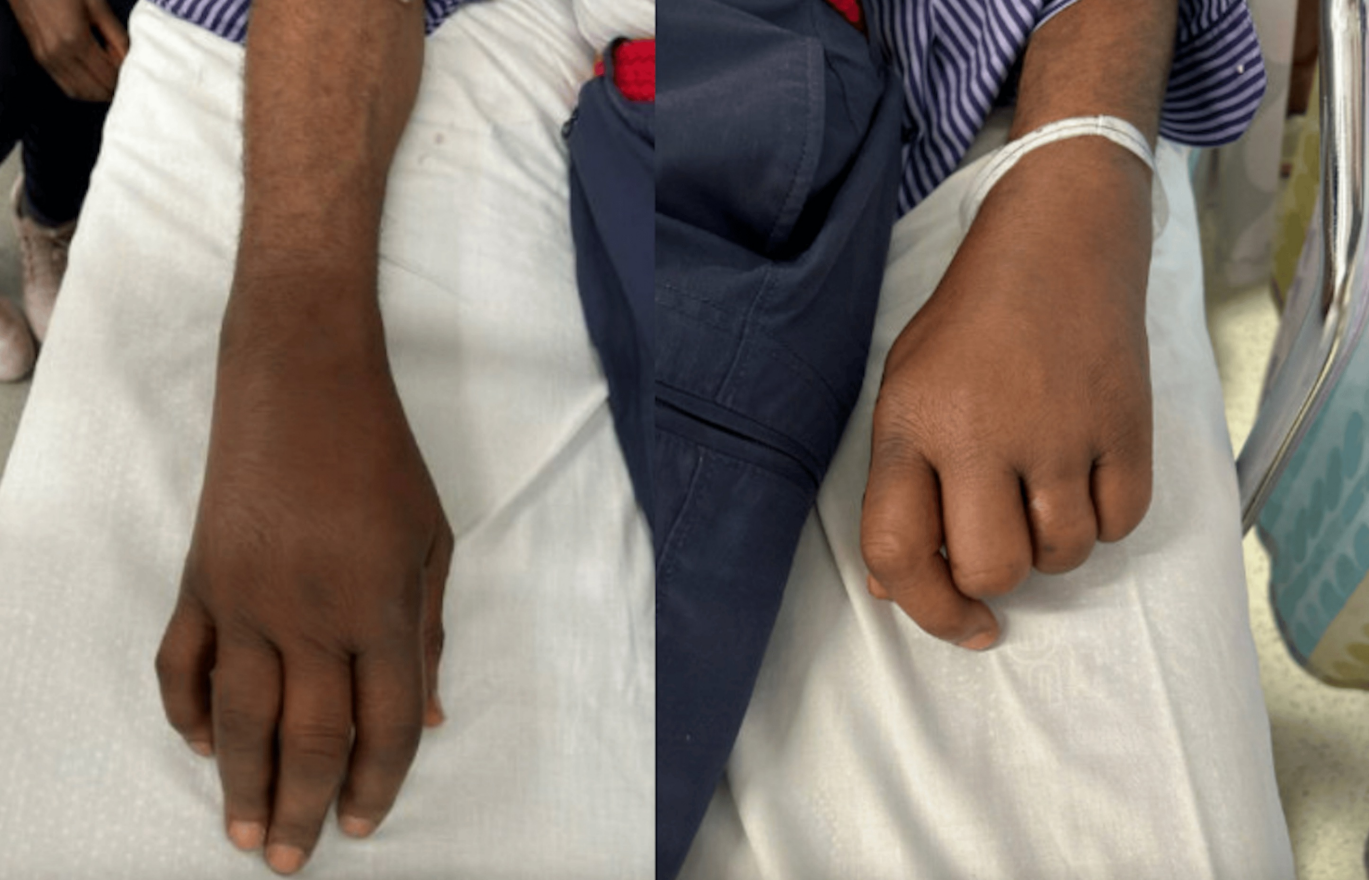
Patient’s right and left wrists and hands on day 0 Bilateral wrists had notable swelling and tenderness that extended to the MCP and PIP joints MCP: metacarpophalangeal; PIP: proximal interphalangeal

**Figure 2:**
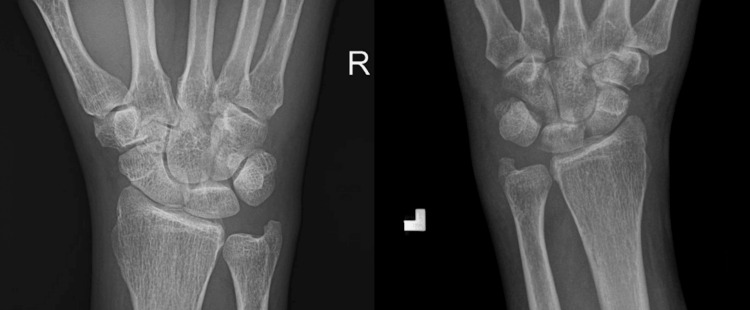
X-rays of left wrist (left) and right wrist (right) on day 0 Bilateral wrists demonstrated diffuse soft tissue swelling around the wrist and dorsal hand. A definite fracture was not identified

On physical examination, the patient’s wrists had notable warmth, tenderness, and swelling, as seen in Figure 1. The tenderness and swelling extended to the metacarpophalangeal (MCP) and proximal interphalangeal (PIP) joints, limiting grip strength. There was no active range of motion of the left wrist, and a limited passive range of motion. The patient had elevated inflammatory markers with C-reactive protein (CRP) at 159.6 mg/L and a white blood cell count of 12.5 x 10^3^/uL upon admission, but he did not have a fever. He was given methylprednisolone 125 mg IV on the day of admission and prednisone 50 mg PO on day one of admission. His X-ray showed soft tissue swelling of the wrist (Figure 2). Urinalysis was mostly unremarkable except for mild proteinuria on day two.

**Figure 3:**
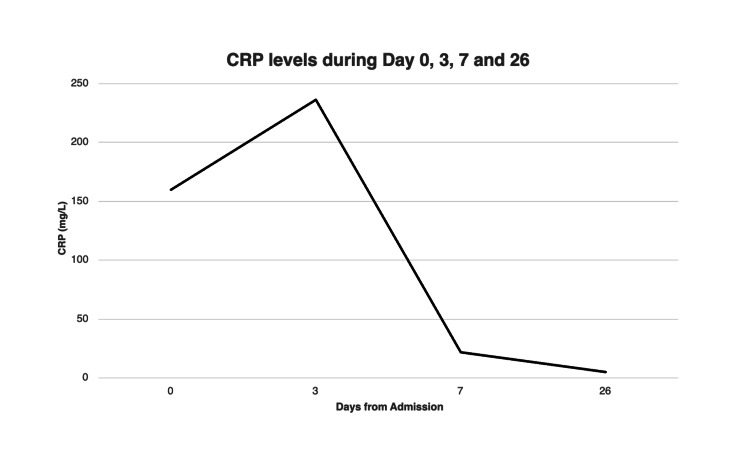
CRP values on days 0, 3, 7, and 26 CRP levels over time (reference range: <10 mg/L). Note the sharp decline post-day three following identification of *N. gonorrhoeae *and therapy adjustment. CRP levels returned to normal by day 26. There was a 90.7% decrease in CRP from day three of admission to 26 days post-admission CRP: C-reactive protein; N. gonorrhoeae: *Neisseria gonorrhoeae*

The patient underwent left wrist arthrocentesis on day one, which came back as a dry tap. The following day, he was admitted to the department of medicine, and an MRI was scheduled. Another left wrist arthrocentesis was attempted on day three, which resulted in another dry tap without synovial fluid, limiting the diagnostic capability by way of evaluating for septic arthritis. Due to concern for crystal arthropathy, the patient was started on anakinra 100 mg daily for three doses total and prednisone (10 mg) for two days. Despite high-dose steroids, his CRP peaked at 236 mg/L on day three post-admission, raising suspicion of left wrist septic arthritis due to worsening pain, restricted motion, and elevated markers (Figure 3). Bilateral wrist MRI indicated subtle erosive changes of the right wrist with global synovial enhancement of the flexor and extensor tendons with small intrasheath fluid but no abscess. Additionally, there were subtle erosive changes of the left wrist with joint space narrowing and small wrist joint fluid with a small fluid pocket. Table 1 presents the complete blood count values on days zero, one, three, seven, and 26

**Table 1:** Complete blood count (CBC) on days 0, 1, 3, 7, and 26 Notable reduction in white blood cell count during the first week of admission upon administration of ceftriaxone was observed H: high value (above reference range); L: low value (below reference range)

Component	Reference range	Day 0	Day 1	Day 3	Day 7	Day 26
White blood cell count	4.2 - 9.1 x 10^3^/uL	12.5 (H)	13.8 (H)	11.7 (H)	7.1	7.0
Red blood cell count	4.60 - 6.00 x 10^6^/uL	4.40 (L)	4.61	4.56 (L)	4.20 (L)	4.13 (L)
Hemoglobin	13.7 - 17.5 g/dL	12.8 (L)	13.4 (L)	13.2 (L)	12.2 (L)	12.0 (L)
Hematocrit	40 - 51%	38.7 (L)	41.3	41.6	38.6 (L)	38.1 (L)
Platelet count	150 - 400 x 10^3^/uL	593 (H)	606 (H)	579 (H)	418 (H)	329
Lymphyocytes	22 - 53%	7 (L)	4 (L)	9 (L)	15 (L)	27
Monocytes	5 - 12%	8	1 (L)	7	9	11
Eosinophils	1 - 7%	0 (L)	0 (L)	1	3	12 (H)
Basophils	0 - 1%	0	0	0	1	1

**Figure 4:**
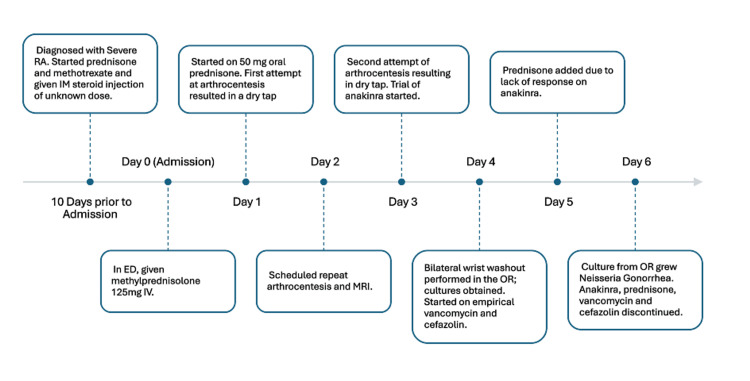
Timeline of patient's clinical course (starting 10 days prior to admission) This timeline outlines the patient's clinical course, beginning 10 days prior to admission. Diagnostic efforts were complicated by unsuccessful arthrocentesis attempts on day one and day three, both yielding dry taps. A definitive diagnosis was achieved following operative intervention and culture results ED: emergency department; IV: intravenous; MRI: magnetic resonance imaging; OR: operating room

On day four, the patient was taken to the operating room (OR) for bilateral wrist washout and arthrocentesis. No frank pus was observed during the operation; rather, a dark, murky fluid and bilateral tenosynovitis were noted. During the OR washout, wrist tissue cultures were taken. ID started empirical vancomycin and cefazolin while awaiting culture results. At this time, the patient showed minimal response to anakinra, and prednisone 10 mg PO was added. On day six, both biopsy and tissue culture grew *N. gonorrhoeae* on the left wrist sample but not in the right wrist sample. Anakinra was discontinued, prednisone was stopped, vancomycin and cefazolin were discontinued, and IV ceftriaxone 2 g was started every 24 hours for two weeks. Further history obtained from the patient confirmed an unprotected sexual encounter two to three months before admission, likely the cause of the gonococcal infection. The patient experienced slight improvements in hand and wrist pain and swelling, with the right wrist improving more than the left wrist. Inflammatory markers quickly down-trended, with CRP levels reaching 21.9 mg/L down from a peak of 236 mg/L in the emergency department one week prior. The full timeline of the patient’s clinical course is presented in Figure 4.

One and a half weeks after discharge, the left wrist continued to show slow improvement compared to the right wrist, which was consistent with gonorrhea growing from only the left wrist in the tissue culture. However, the patient reported daily improvement in pain and had minimal tenderness on physical exam. He completed the two-week ceftriaxone antibiotic course and was referred to hand occupational therapy times times a week for 12 weeks, focusing on passive and active range of motion. Forty-one days post-op, the patient reported significant improvement of the right wrist, with improving but persistent swelling and pain of the left wrist. He was advised to continue with in-house occupational therapy with a focus on digital range of motion, pronation and supination, and wrist flexion.

## Discussion

Although septic arthritis is a common manifestation of DGI, it is not generally associated with a gonococcal infection. Gonococcal septic arthritis typically presents with asymmetric, migratory polyarthralgia accompanied by systemic symptoms such as fever and chills [9]. However, this case highlights the diagnostic challenges posed by atypical presentations, particularly in older adults who fall outside the demographic typically associated with gonorrhea. 

In 2023, diagnosis of gonorrhea was relatively uncommon in ages above 65, making up approximately 0.6% of the total cases compared to the 20-24-year-old age range, which made up the largest percentage of total cases at 23.7% [10]. The low prevalence of gonorrhea in older adults often excludes them from routine STI screening, delaying diagnosis of DGI [11]. However, DGI in patients aged at least 39 years with or without urogenital symptoms has been noted in recent reports, with a higher proportion of older US adults affected by DGI compared to 2023 per CDC [10,12,13]. This discrepancy could be due to selection bias and concurrent conditions within these isolated populations, but raises suspicion for undiagnosed DGI in older demographics. The suspicion is heightened when considering racial epidemiologic data on gonorrhea and STIs. While acknowledging that race is a social construct, African Americans are disproportionately affected by gonorrhea and other STIs in the US, per the CDC [3,14,15].

In our case, diagnostic efforts were complicated by multiple arthroscopies that resulted in dry taps, delaying the discovery of *N. gonorrhoeae*, which was ultimately identified via tissue biopsy obtained during bilateral wrist irrigation and debridement. This was further complicated by the absence of any known unprotected sexual history until after the procedure. Additionally, the patient’s use of high-dose corticosteroids and methotrexate likely blunted systemic signs of infection, such as fever, despite markedly elevated inflammatory markers (CRP and WBC count). By reducing systemic symptoms associated with sepsis, corticosteroids and other immunosuppressive drugs may lead providers to interpret severe joint pain, tenderness, and swelling as an exacerbation of inflammatory arthritis [16]. The initial differential diagnosis included acute RA exacerbation, remitting seronegative symmetrical synovitis with pitting edema, seronegative spondyloarthritis, and crystal arthropathies such as gout and pseudogout, given the acute onset of severe polyarticular pain and swelling; however, these were eventually ruled out due to a lack of response to high-dose corticosteroids. 

Laboratory diagnosis of gonorrhea can be established through a combination of clinical symptoms, bacterial culture, and synovial fluid cultures from mucosal sites, and, if available, nucleic acid amplification technologies (NAAT). *N. gonorrhoeae* is difficult to culture as only 50% of cases yield positive results from blood and synovial fluid, but it can be used to assess antimicrobial resistance (AMR) [5,9,17]. Cultures of mucosal swabs do show improvement over blood and synovial fluid, being positive in 80% of cases [9]. While urine gonorrhea NAAT tests are approximately 99% sensitive and specific in men, making it the more efficacious test, it cannot assess AMR [9,17].

Earlier utilization of NAAT following the initial dry tap could have expedited the diagnosis and initiation of targeted therapy. NAAT not only can reduce time to diagnosis when compared to cultures, but also detects coinfecting pathogens such as *Chlamydia trachomatis* [1]. However, negative urine NAATs in culture-confirmed gonococcal arthritis cases have been reported [9]. Even without confirmation via NAAT or culture, initiation of broad antibiotic treatment could have ameliorated lingering symptoms. A recent study suggests that initiating antibiotic treatment based on high clinical suspicion in patients with negative synovial fluid cultures yields outcomes comparable to those with positive cultures [18]. Nonetheless, improper use of ceftriaxone or cephalosporin antibiotics poses a significant risk of contributing to the emergence of ceftriaxone-resistant *N. gonorrhoeae. *

## Conclusions

This report underscores the importance of maintaining a high index of suspicion for DGI, even in older adults who fall outside the traditional demographic associated with STIs. A detailed sexual history should be obtained in all patients presenting with atypical or severe joint symptoms, regardless of age or assumed risk. Timely recognition of DGI is critical, especially in cases with subtle or absent systemic symptoms, and can be mitigated through early use of NAAT to aid in diagnosis when cultures are inconclusive. Bilateral and symmetric joint involvement of gonococcal septic arthritis is rare and may require surgical tissue culture as a diagnostic step. Clinicians should also maintain suspicion of DGI in patients with unusual presentations, especially in immunosuppressed individuals or those with a history of unprotected sexual encounters.
